# China's reform of the regulatory system for medical products and its impact

**DOI:** 10.1093/nsr/nwz001

**Published:** 2019-02-25

**Authors:** Ruilin Song, Guowei Sang, Meiyu Geng, Hualiang Jiang

**Affiliations:** 1 Research Center of National Drug Policy and Ecosystem, China Pharmaceutical University, China; 2 China Pharmaceutical Innovation and Research Development Association, China; 3 Shanghai Institute of Materia Medica, Chinese Academy of Sciences, China

On 9 August 2015, the State Council of the People's Republic of China issued ‘The Opinions on Reforming Review and Approval Process for Drugs and Medical Devices' (GUOFA [2015] No. 44). This important policy document not only provides the guideline for creating a more scientific and efficient regulatory process for drugs and medical devices, but also initiates the reform of the administration system for medical products in China. In October 2017, the general office of the Communist Party of China Central Committee and the general office of the State Council jointly announced ‘Opinions on Deepening the Reform of Review and Approval System and Encouraging Innovation of Drugs and Medical Devices', which systematically gives specific provisions for the reform of China's drug and medical-device regulatory system. Since 2017, the China Food and Drug Administration (CFDA) has become a member of the International Council for Harmonization (ICH) and has begun to bring international standards into the drug regulatory process in China. After 3 years of efforts, tremendous progress has been made in the development of innovative drugs: the number of new drug applications (NDAs) is increasing; many new drugs are launched to the market; more and more Chinese medical products meet the international standards; and the pharmaceutical industry makes more contributions to the economic growth in China.

With the deepening reform of the review and approval system, previous restrictions on drug and medical-device innovation have been removed, resulting in an increased number of investigational new drug (IND) applications annually. In 2015, there were 70 class I chemical drug IND applications (in terms of active pharmaceutical ingredients). The number increased to 89 in 2016, 143 in 2017 and 132 by the end of November 2018. Moreover, the approval process of foreign drugs has also accelerated. In 2018, more than 30 imported therapeutic agents were approved, showing an improved efficiency of the review and approval system due to the reform. Since the timeline of drug approval is significantly shortened, Chinese patients can take foreign medications sooner than before. In 2018, the number of approved NDAs hit a historical high in China. Innovation makes its great impact on drug development. Since 2008, the National Science and Technology Major Program (MP) ‘Key New Drug Discovery and Development Project' has supported many innovative medical products to participate in international collaboration and competition. By the end of 2018, 37 new class I drugs were approved for marketing. Among them, Roxadustat is the first first-in-class innovative drug developed in China. As the first Chinese humanized anti-programmed-death-1 (PD-1) monoclonal antibody, JS001 entered the market in 2018, in competition with a foreign counterpart marketed in China 4 years ago. In addition, MP-supported enterprises have developed 170 medical products approved by the US Food and Drug Administration (FDA) and launched international multi-center clinical trials for nearly 100 INDs. Among these medical products developed by Chinese enterprises, 4 vaccines and 21 chemical drugs received the World Health Organization Prequalification. International collaboration between Chinese and foreign organizations is strengthening. Many pioneer enterprises, such as Innovent, BeiGene, Hengrui Pharma, Hutchison MediPharma and Ascentage Pharma have initiated clinical trials in foreign countries.

Through technology transfer and/or product authorization, Chinese enterprises are actively cooperating with multinational pharmaceutical corporations to make a presence in the international market. Chinese pharmaceutical companies are increasing their research and development (R&D) expenditure that occupies a growing proportion of revenues. In 2017, Hengrui Pharma invested 1.76 billion yuan in R&D, accounting for 12.7% of its sales and an increase of 48.5% compared to 2016. Fosun Pharma spent 1.53 billion yuan on drug development in 2017, reflecting about 8.3% of its sales and an increase of 32.4%. The pharmaceutical industry is growing at a rapid pace and playing a more and more important role in the nation's economy. Pharmaceutical-industry-added value above the designated size kept a double-digit annual growth from 2011 to 2018—higher than the average value of the whole industrial sector by 4–6%. The contribution of the pharmaceutical industry to the gross domestic product (GDP) was increased from 2.3% (in 2011) to 3.4% (in the first half of 2018). Obviously, innovation of the indigenous pharmaceutical industry is becoming a powerful driving force for the rapid economic development in China.

